# Supplementary material for: The Target ALS Global Natural History Study: Cross-platform proteomics to accelerate biofluid biomarker and drug target discovery in amyotrophic lateral sclerosis
Source: medRxiv. 2026 Jun 23:2026.06.13.26355379. Preprint. [Version 1] doi: 10.64898/2026.06.13.26355379 (PMC13321115; doi:10.64898/2026.06.13.26355379)
Supplement: 1 [file NIHPP2026.06.13.26355379v1-supplement-1.pdf]

medRxiv preprint doi: https://doi.org/10.64898/2026.06.13.26355379; this version posted June 23, 2026. The copyright holder for this preprint (which was not certified by peer review) is the author/funder, who has granted medRxiv a license to display the preprint in perpetuity.
It is made available under a CC-BY 4.0 International license .

Supplementary Figures

Supplementary Figure 1. CSF and plasma protein detection by 35plex TMTpro-MS
For CSF, a total of 2,875 proteins across 14 TMT sets were detected (A). After quality control to include CSF samples with at least 50% of proteins having quantification, 2,120 and 2,163 proteins among our sALS and healthy control cohorts, respectively were included for comparative analysis (B). For plasma, a total of 1,118 proteins were detected (C). Removel of proteins absent in at least 50% of cases yielded 688 and 715 plasma proteins that were compared between sALS and control, respectively.

Supplementary Figure 2. Tissue enrichment of CSF and plasma differentially expressed proteins (DEPS) in sALS.
DEPs were assigned to tissue-associated functional categories based on Gene Ontology Biological Process (GO BP) annotations and displayed as platform-specific proportions (100% stacked bars; 35-plex TMTpro-MS and Olink HT), with total DEP counts indicated above each bar. Upregulated and downregulated DEPs in CSF are shown in (A) and (B), and upregulated and downregulated DEPs in plasma in (C) and (D), respectively. The Olink HT platform predominantly captured muscle/atrophy-associated proteins across both biofluids, while TMTpro-MS captured a substantial proportion of CNS-associated proteins in CSF (46.6% of downregulated and 16.0% of upregulated CSF DEPs).

Supplementary Figure 3. Plasma proteome coverage of 35plex TMTpro-MS versus Olink HT
Kernel density estimate (KDE) plots matching estimated plasma protein concentrations [32] to A) all plasma proteins detected in TMTpro-MS and Olink and B) plasma proteins detected by only one method. Differences in protein concentration between platforms were tested using a two-sided Wilcoxon rank-sum test.

Supplementary Tables

Supp Table 1. Target ALS CSF cohort.
ALSFRS-R- amyotrophic lateral sclerosis functional rating score-revised, STD- standard deviation

Supp Table 2. Target ALS plasma cohort.
ALSFRS-R- amyotrophic lateral sclerosis functional rating score-revised, STD- standard deviation, N/A- not available

Supp Table 3. CSF Proteomic Comparisons for 35-plex TMT-MS and Olink (full list).

medRxiv preprint doi: https://doi.org/10.64898/2026.06.13.26355379; this version posted June 23, 2026. The copyright holder for this preprint (which was not certified by peer review) is the author/funder, who has granted medRxiv a license to display the preprint in perpetuity.
It is made available under a CC-BY 4.0 International license .

HC- healthy control, sALS- sporadic amyotrophic lateral sclerosis, STD- standard deviation, CV- coefficient of variation, adj FDR- adjusted false discovery rate, log2FC- log2 fold-change

Supp Table 4. Plasma Proteomic Comparisons for 35-plex TMT-MS and Olink (full list).
HC- healthy control, sALS- sporadic amyotrophic lateral sclerosis, STD- standard deviation, CV- coefficient of variation, adj FDR- adjusted false discovery rate, log2FC- log2 fold-change

Supp Table 5. List of proteins detected in TMTpro-MS, list of proteins detected in only control or sALS.
TMTpro-MS- 35-plex isobutyl proline tandem mass tag mass spectrometry, HC- healthy control, sALS- sporadic amyotrophic lateral sclerosis CSF- cerebrospinal fluid

Supp Table 6. CSF and plasma DEPs identified by 35plex TMTpro-MS analyses
DEPs- differentially expressed proteins, TMTpro-MS- 35-plex isobutyl proline tandem mass tag mass spectrometry, HC- healthy control, sALS- sporadic amyotrophic lateral sclerosis CSF- cerebrospinal fluid, adj FDR- adjusted false discovery rate, log2FC- log2 fold-change

Supp Table 7. Gene ontology analysis of CSF and plasma DEPs identified by 35plex TMT-MS analysis.
DEPs- differentially expressed proteins, CSF- cerebrospinal fluid, HC- healthy control, sALS- sporadic amyotrophic lateral sclerosis

Supp Table 8. Common and unique CSF proteins detected by 35plex TMTpro-MS and Olink HT.
CSF- cerebrospinal fluid, HC- healthy control, sALS- sporadic amyotrophic lateral sclerosis, TMTpro-MS- 35-plex isobutyl proline tandem mass tag mass spectrometry

Supp Table 9. Common and unique CSF DEPs detected by 35plex TMTpro-MS and Olink HT.
DEPs- differentially expressed proteins, CSF- cerebrospinal fluid, HC- healthy control, sALS- sporadic amyotrophic lateral sclerosis, TMTpro-MS- 35-plex isobutyl proline tandem mass tag mass spectrometry

Supp Table 10. Common and unique plasma proteins detected by 35plex TMTpro-MS and Olink HT
HC- healthy control, sALS- sporadic amyotrophic lateral sclerosis, TMTpro-MS- 35-plex isobutyl proline tandem mass tag mass spectrometry

Supp Table 11. Common and unique plasma DEPs detected by 35plex TMTpro-MS and Olink HT
DEPs- differentially expressed proteins, HC- healthy control, sALS- sporadic amyotrophic lateral sclerosis, TMTpro-MS- 35-plex isobutyl proline tandem mass tag mass spectrometry

Supp Table 12. WU CSF validation cohort
CSF- cerebrospinal fluid, STD- standard deviation

Supp Table 13. WU plasma validation cohort
STD- standard deviation
